# Response of broilers to supplementation of branched-chain amino acids blends with different valine contents in the starter period under summer conditions

**DOI:** 10.5713/ajas.19.0828

**Published:** 2020-02-25

**Authors:** Canan Kop-Bozbay, Ahmet Akdag, Helin Atan, Nuh Ocak

**Affiliations:** 1 Department of Animal Science, Faculty of Agriculture, Eskisehir Osmangazi University, 26480, Eskisehir, Turkey; 2 Department of Animal Science, Faculty of Agriculture, Ondokuz Mayis University, 55139, Samsun, Turkey

**Keywords:** Poultry, Amino Acids, Productive Performance, Carcass Yield, Digestive System, Muscle Growth, Meat Quality

## Abstract

**Objective:**

The objectives of this study were to compare the effects of normal and low protein content (PC) of starter diet supplemented or not with blends of branched-chain amino acids (BCAAs) on growth performance of broilers under summer conditions and to investigate whether these effects altered some quality traits and the characteristics of gastrointestinal tract.

**Methods:**

A total of 768 mixed-sex broiler chicks (Ross 308, one-d-old) with an average initial body weight (BW) of 47.6±1.03 g were allocated into six treatments with four replications in 2×3 factorial arrangement. Factors were: PC, normal (N, 22% to d 15); and low (L, 20% to d 15); and added BCAA blends, L-leucine, L-isoleucine, and L-valine at zero (0L:0I:0V); 1.0, 0.25, and 0.25 (4L:1I:1V); or 1.0, 0.25, 0.75 (4L:1I:3V) g/kg of diet. Hence, six dietary treatments were named as N0L:0I:0V, N4L:1I:1V, N4L:1I:3V, L0L:0I:0V, L4L:1I:1V, and L4L:1I:3V. Average indoor temperature and humidity were 32.8°C±1.7°C and 61.1% ±4.12%, respectively.

**Results:**

BW, feed conversion ratio (FCR) and carcass weight were not affected by PC, BCCA and their interaction (p>0.05). The L diets decreased the water holding capacity of the breast (p = 0.002) and thigh (p = 0.050) meats and dressing percentage (p = 0.005) compared to the N diets. The 4L:1I:1V diet decreased breast yield compared to the 0L:0I:0V diets (p = 0.041). The effect of PC on feed intake, mortality and gastrointestinal trait weight were depended on the L:I:V ratios under summer conditions due to interactions between factors (p<0.05). The FI and mortality of L4L:1I:1V broilers were lower than those of N4L:1I:1V birds (p<0.05).

**Conclusion:**

It was concluded that the blends of BCAAs used failed to improve performance and to promote breast yields, because diets with normal or with reduced protein supplemented or not with BCAAs up to d 15 produced a similar BW and FCR in broilers raised in hot-climate conditions.

## INTRODUCTION

Optimal growth rate, feed intake (FI) and thereby feed conversion ratio (FCR) in broiler chickens require dietary crude protein (CP) with optimum amino acids (AAs) composition during early stages of growth. Such dietary protein sources are the most expensive ingredients for poultry diets. Therefore, one of the greatest challenges to the efficient production of broilers is lack of AAs resulting in a delay in the development of gastrointestinal tract (gut) and proliferation of muscle cells associated with early protein supply [1]. Indeed, the final growth rates of broilers are directly proportional to the early growth rate of its skeletal muscles and especially gut [2]. Therefore, there is a trend towards the reduction of CP content (PC) in broiler diets [1,3,4]. Commercial branched-chain amino acids (BCAAs) supplements may allow the dietary CP level to reduce [5–7], because leucine, isoleucine and valine are not merely three of the nine essential AAs for poultry, but also are proteinogenic AAs that are regulated protein synthesis in a variety of tissues [8]. As such, the BCAAs supplements may improve the development of gut and proliferation of muscle cells during early stages of growth while overcoming some problems such as cost of production and soil pollution associated with the protein nutrition [5,9,10].

Effects of exogenous nutrients on the development of gut and muscle in broiler chicks may be more effectively examined during early stages of growth [8]. At this period, low-CP diets supplemented or not with BCAAs either as single or combined mixtures for broilers have, therefore, been the subject of extensive studies [5–7]. However, there are discrepancies among the findings of these studies. Because there are interactions among the BCAAs, an increase in its activity (i.e. due to leucine excess) increases the catabolism of the three BCAAs, in case of valine and isoleucine deficiencies a high dietary leucine content may have caused an increase in valine and isoleucine requirements of broilers [5,7,11]. Moreover, this effect of leucine is more potent on valine than on isoleucine in environmentally controlled houses [5]. Therefore, the anti-proteolytic or anti-catabolic effect of leucine is promoted in the presence of valine and isoleucine [12]. Although feeding low-CP diets while adding more crystalline BCAAs (in particular valine and isoleucine) than that required to broiler diets during early ages has gained importance [6,11,13,14], the response of broilers to blends of BCAAs with different valine contents is not well defined in the hot-climatic conditions.

A reduction in FI of broilers reared in hot-climate region could change the partition and utilization of AAs in the metabolism of birds due to reduced protein intake [10,11]. Dietary valine has decreased the negative effect of feeding low-CP diets in broiler chickens through the beneficial effect on protein accretion and intestinal morphology [6,13]. Therefore, we hypothesised that during the first two weeks, the mixture of BCAAs with increased content of valine may provide an opportunity to reduce the CP of the broiler starter diet and to mitigate the effects of heat stress from summer conditions on growth performance. Consequently, these may change carcass yield, the characteristics of non-carcass parts and meat quality. Accordingly, the objectives of this study were to compare the effects of normal and low-PC of starter diet supplemented or not with blends of BCAAs on growth performance of broilers under summer conditions and to investigate whether these effects altered some quality traits of breast and thigh (leg quarters) meats, liver colour values and the characteristics of whole gut and its some segments.

## MATERIALS AND METHODS

### Animal care and study site

This study, conducted between June and July 2017, was carried out at the Research Farm of Agricultural Faculty of Eskisehir Osmangazi University in Eskisehir, Turkey (39°45′42″ N and 30°28′40″ E, and altitude of 813 m above sea level). The study was approved by the local Ethics Committee for Experimental Animals, which ascertained that the experiment was not an unnecessary repetition of previous experiments (case number: HAYDEK-582/2017).

### Animals, diets, and management

A total of 768 one-d-old chicks (Ross 308) obtained from a commercial hatchery (Ross Breeders, Inegol, Turkey) were weighed. The sex of each chick was determined according to the difference in design and size of flight feathers on the wings of males and females in the first days after hatching. Then, they were allocated into six treatments (128 mixed-sex broilers each) according to initial body weight (average 47.6±1.03 g/chick) in 2×3 factorial arrangement. Each treatment had four replicated pens with 32 birds (16 females and 16 males) per pen, which was considered as an experimental unit. Factors were: PC, normal (N, 22% to d 15) and low (L, 20% to d 15), and added blends of BCAAs (BCAA), graded levels of L-leucine (Cas no: 61-90-5), L-isoleucine (Cas no: 72-18-4), and L-valine (Cas no: 73-32-5) were supplemented to the N and L diets at zero (0L:0I:0V), 1.0, 0.25 and 0.25 (4L:1I:1V) or 1.0, 0.25, 0.75 (4L:1I:3V) g/kg of diet, accomplished by adding the blends of these AAs at the expense of an inert filler. Hence, six dietary treatments were named as N0L:0I:0V, N4L:1I:1V, N4L:1I:3V, L0L:0I:0V, L4L:1I:1V, and L4L:1I:3V. Broiler starter diets were formulated according to the management guide for Ross 308 [15] to contain 220 g CP and 3,050 kcal metabolizable energy (ME)/kg in starter diets, and 200 g CP and 3,200 kcal ME/kg in grower diets (Table 1). The content of standardized available AAs in all diets [16] was similar (Table 2). The feeding program had two phases: starter (d 1 to 21) and grower (d 22 to 42). From 16 to 42 days of age, all birds were fed following the relevant feeding program recommended in the management guide for Ross 308 broiler [15].

**Figure 1:**
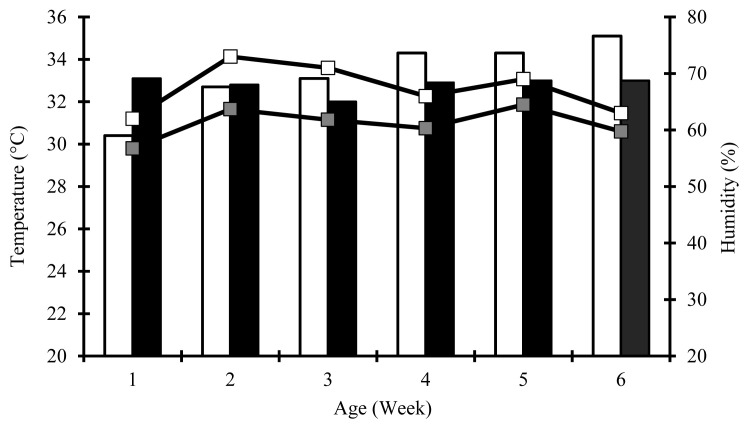
Average weekly temperature (bar) and humidity (line) in the outdoor (□) and indoor (■) during the experiment.

During the entire experiment, all chicks were reared on the floor pens (2.5 m length×1.0 m width×4.5 m height) littered with wood shavings. All feeds (mash form) and water were provided *ad libitum* using red circular poultry feeder plates and automatic drinkers. Artificial light provided by two 125-watt bulb lamps was continuous in the first week and 23 hours afterwards. The experiment was performed under standard husbandry practices, except for ambient temperature. This study was carried out in summer environmental conditions (Figure 1). However, because ambient temperature was 33°C±1°C during the brooding period (the first two weeks), chicks were not exposed to any heat stress. From d 15, all the birds were exposed to high temperature (36°C±1.2°C) and humidity (70%±2.5%) for 3 h from 11:00 to 14:00 every day until the end of the experiment due to outdoor temperature and humidity. However, average weekly temperature and humidity in the indoor during the experiment could be kept at 32.8°C±1.7°C and 61.1%±4.12%, respectively. After the heat challenge, the time required to decrease the temperature from 36°C to 24°C was 1 h 30 min.

### Feed intake and growth performance

The broilers were weighed on a pen basis at periods corresponding to the treatment phases (at 15 and 42 d of age), while FI was recorded on d 7, 14, 21, 28, 35, and 42. These were then used to calculate daily body weight gain (BWG) and FI. Health status of birds was monitored at least twice daily. The gender, number and weight of dead birds found at these inspections were recorded to calculate mortality and FCR (g feed:g gain) corrected for mortality. Therefore, it was made to adjust the data as per numbers of males and females in each pen on weigh days and FCR was calculated by adding the weight of dead birds.

### Carcass characteristics and the size of internal organs

On d 14 and 42, after 10 h fasting and free access to feed, four birds with BW within one standard deviation of the mean treatment weight were slaughtered humanely and chilled at 4°C for 12 h. The influences of gender were not determined on the studied parameters. To determine weights of carcass and metabolically active organs (liver, heart, pancreas, whole gut and its segments) and tissues (breast and thigh muscles), abdominal cavities of all carcasses were opened and then, they were carefully removed and weighed after its contents were cleaned [12,17]. The small intestine was carefully dissected from the mesentery and divided into its segments as duodenum, jejunum and ileum [6]. Carcass yield that expressed as dressing percentage and relative weights of organ and tissue were calculated using the BW just before slaughter of the broilers and thus expressed as a percentage of BW just before slaughter (g/100 g BW).

### Meat quality traits

On d 42, nutritional and visual quality characteristics of skeletal muscles and the colour values of the liver were also determined. The samples from the raw breast and thigh meats were mashed and dry matter (DM; method 930.15), ash (method 942.15), CP (method 990.03) and ether extract (EE, method 920.39) were performed in triplicate according to the approved methods [18]. Proximate analysis results were expressed in wet basis. The Commission Internationale de l’Eclairage L* (lightness), a* (redness), and b* (yellowness) values (CIELab) were measured 24 h after the slaughter using a Minolta CR 300 Chroma Meter (Minolta Camera, Osaka, Japan). Four and three replicate measures on the meat and liver samples, respectively, were made and expressed as the mean CIELab values calculated for each sample [12]. To determine water holding capacity (WHC) of breast and thigh meats, meat cubes of 1 g were rolled with filter paper and put into Eppendorf tubes and centrifuged for 4 min× 1,500 g [19]. The samples removed from the papers were weighed and then, the weight difference between the initial and final weight of samples was calculated. The WHC values were expressed as the percentage of drip loss relative to the initial sample weight.

### Statistical analysis

The experiment was designed as a 2×3 factorial and the data were analyzed using the general linear model procedure in SPSS Release 21.0 (SPSS Inc., Chicago, IL, USA) in the model as follows:

$$Y_{\text{ijk}}=\mu +P_{\text{i}}+B_{\text{j}}+PB_{ij}+e_{\text{ijk}}$$

Whereas *Y*_ijk_ is the feeding effect on birds, *μ* was the mean value, *P*_i_ is an effect of PC, *B*_j_ is the effect of BCAA, *PB*_ij_ is the interaction of the PC and BCAA factors, and *e*_ijk_ is the error value. Data regarding mortality, relative organ weights were analyzed after arcsine transformation [(% data/100)+0.05]^0.5^. Normality and homoscedasticity of these data were verified by Levene’s test (SPSS Inc., USA). The significant differences among means were analyzed by using the Tukey test and were deemed significant at p<0.05.

## RESULTS

### The effect of crude protein content

The BWG, FCR, and carcass weight of broiler chickens were not affected by the PC treatment at 14 and 42 d of age (Table 3). Compared to the N diet, the L diet decreased the dressing percentage (p = 0.002) on d 42 (Table 3), the pancreas weight (p = 0.005, Table 4) and the protein concentration of thigh meat (p = 0.023, Table 5) on d 14. The L diet increased the pancreas weight (p = 0.005) on d 14 (Table 4) and the WHC of the breast (p = 0.002) and thigh (p = 0.050) meats on d 42 (Table 6).

### The effect of the branched-chain amino acids blends

The BWG, FCR, and carcass weight of broiler chickens were not affected by the BCCA treatment at 14 and 42 d of age (Table 3). Breast yield was negatively affected by the 4L:1I:1V diet compared to the 0L:0I:0V diet (p = 0.041; Table 4). At 14 d of age, the DM content of breast meat of the 0L:0I:0V broilers was higher compared to the 4L:1I:3V birds (Table 5). The b* values of the breast (p = 0.040) and thigh (p = 0.035) meats of the 4L:1I:1V broilers were higher than those of the 0L:0I:0V and 4L:1I:3V birds, respectively (p<0.05, Table 6). The liver a* value in the 4L:1I:1V group was higher than those in other groups (p = 0.040). The 4L:1I:3V broilers had higher (p = 0.042) the proventriculus weight compared to the 0L:0I:0V and 4L:1I:1V birds at 14 d of age and had higher (p = 0.047) the jejunum weight compared to the 4L:1I:1V birds on d 42 (Table 7).

### The interaction effect of the studied factors

There were interactions between factors for the weights of the whole gut, jejunum, ileum, large intestine and the DM content of thigh meat at 14 d of age and the FI, mortality and DM content and L* value of thigh meat at 42 d of age (Table 8). The FI and mortality of L4L:1I:1V broilers were lower than those of N4L:1I:1V birds (p<0.05). At 14 d of age, the L4L:1I:1V and L4L:1I:3V diets increased the weight of whole gut compared to the L0L:0I:0V diet, whereas L4L:1I:3V increased the weight of jejunum compared to the L0L:0I:0V, L4L:1I:1V, and N4L:1I:1V (p<0.05). The weights of ileum and large intestine and the DM content of thigh meat of the L0L:0I:0V broilers were higher than those of birds fed other diets, except for the N4L:1I:1V (p<0.05). On d 42, N4L:1I:3V increased the DM content of thigh meat compared to N0L:0I:0V, L0L:0I:0V, and L4L:1I:1V (p<0.05). The broilers fed with N0 diet had lower the thigh meat L* value than that of birds fed with N4L:1I:1V and L0L:0I:0V (p<0.05).

## DISCUSSION

The daily BWG (58.4 g) and FCR (1.84) of the N birds were quite below the standard (65.8 g and 1.68, respectively) expected for mixed-sex broilers under controlled environment conditions [15]. This might be since the heat exposure impairs the intestinal morphology, appetite and consequently performance traits [9–11,20] because the present study was conducted under warm summer conditions (average weekly indoor 32.8°C±1.7°C temperature and 61.1%±4.12% humidity). In agreement with Liu et al [21], our study revealed that the impact of heat stress could be mitigated partially by adjusting PCs of the diets as a nutritional practice. Irrespective of the BCAAs blends used, a higher final BW with similar FI and FCR was achieved in the L broilers. Our results indicated that the BWG, FI, and FCR of the broilers were not influenced by reduced CP [16,21] or the dietary protein profile [9]. Therefore, our findings supported the idea that low CP diets can contribute to the growth of broilers equal to that of normal CP diets [1,3,10].

The effect of the PC on the FI, mortality and gut weights gut weights of the broilers were depended on the L:I:V ratios under summer conditions due to the interaction effect of the factors. Therefore, the BCAAs profile of the low CP diets under warm summer conditions had a beneficial effect on the FI and the livability of the broilers, as reported by Liu et al [21]. These findings indicated that the dietary CP level was probably not so important and the BCAAs profile of the diet was rather a more important factor due to the antagonistic impact between leucine and valine in the diet [5,11,22] or antagonism among BCAAs [11]. Indeed, in the previous studies [5,22], feeding low CP diets with high leucine level decreased the FI of birds, since leucine and valine levels interacted positively with the FI [22]. Also, antagonisms between BCAAs may increase valine requirement, causing broilers to enhance FI [11]. This may explain why there was an interaction between factors for FI. In the present study, however, no benefits were detected for triple the supply of valine in the BCCAs blends in terms of the BWG and FCR in the broilers. Pereira et al [11] observed that there is a positive linear and quadratic effect of valine concentrations on FI and BWG, respectively, in growing male broilers in the hot-climate region.

Our results on the BWG and FCR indicated that the impact of the BCAAs on these variables might not vary by the amount of valine in the BCAAs or the dietary CP, as reported by Miranda et al [7]. However, these results are disagreement with previous some studies [6,22] in which broiler performance was positively affected by valine supplementation. The differences among the previous studies and the present study may be related to the fact that the relationships among BCAAs contribute to the differences in the AA ratios such as valine to lysine [22–24] and/or digestible valine requirements differ for maximum performances and breast yield in broilers fed low-CP diet during the starter phase [11,13,14]. Potença et al [23] reported that a valine to lysine ratio of 66% is sufficient to maximize the performance of broilers from 1 to 14 d of age. However, in a recent study [6], the optimal digestible valine to lysine ratio in the starter phase (0 to 12 d) has been reported as 0.78 for BWG and 0.80 for FCR. Indeed, the valine levels and valine to lysine ratios (0.79, 0.81, and 0.85 for 0L:0I:0V, 4L:1I:1V and 4L:1I:3V groups) of the starter diets in the present study were higher than the recommendations in the previous studies [6,22–24].

In the present study, the final BW was less compared to the standard [15] due to the negative impact of heat stress on the growth performance [10,11], which also may be an explanation for the reduced dressing percentage [3]. Although dietary protein has not affected carcass yield [9] and breast yield [22] in broilers, in some studies, broilers fed the low-CP diet have had lower breast and drumstick yields compared to high- or medium-CP fed birds [3,9]. These reports and our findings indicate that the maximum breast yield requires a normal-CP diet. Furthermore, the valine requirements for the proliferation of muscle cells in broiler chickens at early stages of growth may be altered by high summer temperature [4,13]. These discrepancies may be due to the breed and age of experimental birds, the valine- and isoleucine-to-lysine ratios [6,23,24] and the quality of the ingredients of diets or the severity and duration of high temperature [1,11,25]. Indeed, the previous studies dealing with AAs fortification to low-CP diets in poultry have been carried out using high quality diets under a controlled environment or in confined systems [5–7].

As known, the chemical composition of broiler skeletal muscles can be affected by nutrition. In the present study, the L diet decreased the protein content of thigh meat from broiler chicks on d 14, as was reported by Furlan et al [10]. However, this effect was not reflected in the proximate analysis of breast and thigh meats at the end of the trial. The gut of the newly hatched chick is in a process of development and maturation during the first few weeks of life. The development of gut in broiler chicks can, thus, be affected by the blends of BCAAs having different valine contents during this period, as reported by Norouzian et al [2]. Our results on the development of whole gut may be explained by the fact that the efficiency of dietary protein utilization in poultry depends partly on the features of the gut [9]. The changes in the weights of proventriculus, jejunum and cecum may be due to the proteinogenic functions of the BCAAs [26]. The proteinogenic effects of the BCAAs with different valine contents were not confirmed to the results on the breast and thigh meats, because BCAAs in the present study decreased the breast meat yield, but did not affect thigh meat yield. Therefore, these results indicated that supplementation of valine was not critical in promoting higher carcass and breast weights or yields in broilers, as reported by Agostini et al [24]. These results may be related to negative interactions between supplemental valine and isoleucine [5,11,22].

In poultry, the liver, one of the most active organs, and the studied muscles, the tissue of the highest economic value, are metabolically important organs and tissues. The CIELab values of meats and liver, an indicator of the physiological condition of birds [12], were within previously reported normal range [27]. In the present study, no assessment was done to study tissue energy metabolism. The results concerning the liver, breast and thigh meats indicate that there is a difference in the metabolic energy utilization efficiency of the BCAAs blends with different valine contents in growing birds under summer conditions. Imanari et al [28] reported that the BCAAs profile of broiler diets, especially those with high valine and isoleucine improved the meat quality. One of the main determinants of WHC in meat is post-mortem protein denaturation [29]. A reduction in the WHC causes a decrease in L* and a* values and an increase in b* value of poultry meats [27,30]. In the present study, dietary BCAAs with different valine contents caused a change in the WHC of the studied meats. Although there was an interaction between factors for the L* value of thigh meat [27], the higher WHC of breast and thigh of broiler chickens fed with low-CP starter diet did not affect the L* value of the broiler meats. This indicates that dietary protein and BCAAs levels did not affect the CIELab and WHC values of the meat in broilers fed the same diet throughout the growth phase. The changes in the b* values of breast and thigh meats may be related to a muscle wastage [30]. The increase in the redness value of liver from 4L:1I:1V broilers may be associated with changes in some metabolites and nutrients [26,27] due to participate of BCAAs to some metabolic pathways such as lipolysis, lipogenesis and glycolysis [26].

## CONCLUSION

In the present study, the BCAAs blends supplementation failed to improve FCR as a result of feeding ideal or low protein under summer conditions. Increasing valine in dietary BCAAs blends does not cause a measurable variation in nutritional and visual meat qualities. Also, feeding low-CP diets at the first 14 d of the life of broilers, irrespective of the BCAAs blends, could result in a similar performance. However, supplementation of valine is not critical in promoting higher carcass and breast weights or yields in broilers. The present study can provide a basis for research into the effects of dietary CP supplemented or not with blends of BCAAs on broiler chickens under hot summer conditions. However, to well define the response of broilers to low-CP diets supplemented or not with blends of BCAAs having different valine contents and other essential AAs requires further investigation in normal- and hot-climatic regions.

## Figures and Tables

**Table 1:** Ingredients and calculated nutrient values of the basal diets with normal (N) and low (L) protein1) (as-fed basis)

Items	N starter (1 to 21 d)	L starter (1 to 21 d)	Grower (22 to 42 d)
Ingredients (g/kg)			
Corn (8.2% crude protein)	516.0	587.9	623.0
Soybean meal (46% crude protein)	388.9	325.0	313.0
Soybean oil	59.1	47.3	38.0
Dicalcium phosphate	16.2	17.5	11.0
Limestone	9.8	9.3	7.5
Sodium chloride	4.5	4.7	2.5
L-lysine (78.5%)2)	0.3	1.9	0.5
DL-methionine (99.0%)	1.7	2.4	2.0
L-threonine (98.5%)	-	0.5	-
Vitamin and mineral premix3)	3.5	3.5	2.5
Calculated energy (kcal/kg) and nutrient contents (g/kg)			
Metabolizable energy	3,050	3,050	3,200
Dry matter	880	880	878
Crude protein	220	200	200
Linoleic acid	260	258	248
Ca	10.0	10.0	8.10
Available P	4.7	4.7	4.0
Lysine	12.7	12.8	10.8
Methionine+cysteine	10.4	10.0	9.7

1) Up to 15 d of age, L-leucine, L-isoleucine, and L-valine were added to the starter diets with the N and L protein contents at zero, 1.0, 0.25 and 0.25 or 1.0, 0.25, 0.75 g/kg of diet, respectively. Supplemental BCAA blends were added to the test diets at the expense of the inert filler to derive dietary treatments.

2) To estimate dietary protein, crude protein values of all amino acids were considered [16].

3) Supplied per kilogram of diet: vitamin A, 12,000 IU; vitamin D_3_, 2,400 IU; vitamin E, 30 IU; vitamin K_3_, 2.5 mg; vitamin B_1_, 3.0 mg; vitamin B_2_, 7 mg; nicotine amid, 40 mg; calcium D-pantothenate, 8.0 mg; vitamin B_6_, 4.0 mg; vitamin B_12_, 0.015 mg; folic acid, 1 mg; D-biotin, 0.045 mg; vitamin C, 50 mg; choline chloride, 270 mg; Mn, 80 mg; Fe, 40 mg; Zn, 60 mg; Cu, 5 mg; Co, 0.1 mg; I. 0.4 mg; Se, 0.15 mg.

**Table 2:** Calculated available amino acid values of experimental diets

Amino acids (g/kg)1)	N0L:0I:0V2)	N4L:1I:1V2)	N4L:1I:3V2)	L0L:0I:0V2)	L4L:1I:1V2)	L4L:1I:3V2)
Lysine	12.70	12.70	12.70	12.80	12.80	12.80
Methionine	4.70	4.70	4.70	4.70	4.70	4.70
Methionine+cysteine	8.30	8.30	8.30	8.30	8.30	8.30
Arginine	14.20	14.20	14.20	14.20	14.20	14.20
Phenylalanine	1.03	1.03	1.03	1.03	1.03	1.03
Tyrosine	8.40	8.40	8.40	8.40	8.40	8.40
Threonine	8.10	8.11	8.11	8.10	8.11	8.11
Tryptophan	2.90	2.90	2.90	2.90	2.90	2.90
Leucine	18.70	19.70	19.70	18.70	19.70	19.70
Isoleucine	9.00	9.25	9.25	9.00	9.25	9.25
Valine	10.00	10.25	10.75	10.00	10.25	10.75

1) The available amino acids contents of all diets were calculated basing the values tabulated for individual feedstuffs [16].

2) N0L:0I:0V, the starter diets (22% to d 15) with the normal protein content (PC), added L-leucine, L-isoleucine, and L-valine at zero (0L:0I:0V); N4L:1I:1V, the starter diets with the normal PC added L-leucine, L-isoleucine, and L-valine at 1.0, 0.25, and 0.25 (4L:1I:1V) g/kg of diet; N4L:1I:3V, the starter diets with the normal PC added L-leucine, L-isoleucine, and L-valine at 1.0, 0.25, and 0.75 (4L:1I:3V) g/kg of diet; L0L:0I:0V, the starter diets (20% to d 15) with the low PC added L-leucine, L-isoleucine, and L-valine at zero; L4L:1I:1V, the starter diets with the low PC added L-leucine, L-isoleucine, and L-valine at 1.0, 0.25, and 0.25 (4L:1I:1V) g/kg of diet; L4L:1I:3V, the starter diets with the low PC added L-leucine, L-isoleucine, and L-valine at 1.0, 0.25, and 0.75 (4L:1I:3V) g/kg of diet.

**Table 3:** The growth performance, mortality, carcass weight and dressing percentage of broilers fed starter diets with different levels of protein and blends of branched-chain amino acids under hot summer conditions

Variable	PC1)		BCAA1)			SEM	Main effect of		
		
	N	L	0L:0I:0V	4L:1I:1V	4L:1I:3V		PC	BCAA	PC×BCAA
On 14 d of age									
Body weight gain (g/bird/d)2)	26.24	25.97	26.38	26.59	26.07	0.280	ns	ns	ns
Feed intake (g/bird/d)	39.26	38.75	39.25	39.02	38.74	0.237	ns	ns	ns
Feed conversion ratio (g feed:g gain)	1.49	1.49	1.48	1.46	1.48	0.008	ns	ns	ns
Mortality (%)	1.30	1.82	2.73	1.17	1.17	0.459	ns	ns	ns
Carcass weight (g)3)	274.05	276.11	284.06	277.12	273.20	5.750	ns	ns	ns
Dressing percentage (%)	62.57	63.55	63.87	62.92	62.31	0.400	ns	ns	ns
On 42 d of age									
Body weight gain (g/bird/d)2)	58.48	58.36	59.06	58.43	57.76	0.434	ns	ns	ns
Feed intake (g/bird/d)	107.90	107.98	109.24	106.79	107.80	1.046	ns	ns	*
Feed conversion ratio (g feed:g gain)	1.84	1.85	1.84	1.82	1.86	0.016	ns	ns	ns
Mortality (%)	7.03	7.03	7.81	6.64	5.85	0.834	ns	ns	*
Carcass weight (g)3)	2,057.5	2,016.2	2,048.6	2,055.0	2,009.2	25.51	ns	ns	ns
Dressing percentage (%)	78.41^a^	77.09^b^	77.70	77.73	77.83	0.199	**	ns	ns

SEM, standard error of the mean.

1) PC, crude protein content of starter diets; BCAA, added blends of branched-chain amino acids; N, starter diet (22% to d 15) formulated with normal crude protein; L, starter diet formulated (20% to d 15) with low crude protein; 0L:0I:0V, diet without of BCAA blends; 4L:1I:1V, diet added L-leucine, L-isoleucine, and L-valine at 1.0, 0.25, and 0.75 g/kg; 4L:1I:3V, diet added L-leucine, L-isoleucine, and L-valine at 1.0, 0.25, and 0.75 g/kg.

2) The values are means of the four replicates with 32 birds per treatment.

3) The values are means of the four replicates with two birds per treatment.

ab Within a row, means with different superscripts differ significantly (p<0.05).

ns, nonsignificant; p>0.05;

* p<0.05;

** p<0.01.

**Table 4:** The relative weight of some organs and meats (g/100 g BW) of broilers fed starter diets with different levels of protein and blends of branched-chain amino acids under hot summer conditions

Variable1)	PC2)		BCAA2)			SEM	Main effect of		
		
	N	L	0L:0I:0V	4L:1I:1V	4L:1I:3V		PC	BCAA	PC×BCAA
On 14 d of age									
Heart	0.70	0.71	0.69	0.73	0.69	0.016	ns	ns	ns
Liver	3.16	3.37	3.21	3.28	3.31	0.069	ns	ns	ns
Pancreas	0.37^b^	0.42^a^	0.37	0.42	0.40	0.010	**	ns	ns
Breast	15.14	14.78	15.68	14.60	14.60	0.400	ns	ns	ns
Thigh	11.32	11.62	11.58	11.68	11.24	0.078	ns	ns	ns
On 42 d of age									
Heart	0.51	0.52	0.52	0.54	0.49	0.016	ns	ns	ns
Liver	1.83	1.77	1.92	1.71	1.77	0.043	ns	ns	ns
Pancreas	0.17	0.18	0.18	0.17	0.18	0.007	ns	ns	ns
Breast	23.82	22.92	24.34^a^	22.50^b^	23.24^a^^b^	0.163	ns	*	ns
Thigh	16.24	16.10	16.14	16.06	16.32	0.083	ns	ns	ns

SEM, standard error of the mean.

1) The values are means of the four replicates with two birds per treatment.

2) PC, crude protein content of starter diets; BCAA, added blends of branched-chain amino acids; N, starter diet (22% to d 15) formulated with normal crude protein; L, starter diet formulated (20% to d 15) with low crude protein; 0L:0I:0V, diet without of BCAA blends; 4L:1I:1V, diet added L-leucine, L-isoleucine, and L-valine at 1.0, 0.25, and 0.75 g/kg; 4L:1I:3V, diet added L-leucine, L-isoleucine, and L-valine at 1.0, 0.25, and 0.75 g/kg.

a,b Within a row, means with different superscripts differ significantly (p<0.05).

ns, nonsignificant; p>0.05;

* p<0.05;

** p<0.01.

**Table 5:** The nutrient contents of breast and thigh meats of broilers fed starter diets with different levels of protein and blends of branched-chain amino acids under hot summer conditions

Variable1)	PC2)		BCAA2)			SEM	Main effect of		
		
	N	L	0L:0I:0V	4L:1I:1V	4L:1I:3V		PC	BCAA	PC×BCAA
On 14 d of age									
Breast meat									
Dry matter	26.76	27.84	29.09^a^	26.78^ab^	26.04^b^	0.459	ns	*	ns
Protein	21.58	22.37	21.69	22.56	21.67	0.335	ns	ns	ns
Ether extract	0.77	0.90	0.97	0.66	0.84	0.138	ns	ns	ns
Ash	1.07	1.24	0.97	1.22	1.28	0.081	ns	ns	ns
Thigh meat									
Dry matter	24.74	25.16	24.33	25.51	25.01	0.295	ns	ns	*
Protein	21.60^a^	20.15^b^	20.07	21.70	20.85	0.293	*	ns	ns
Ether extract	2.38	2.21	2.44	2.22	2.23	0.197	ns	ns	ns
Ash	1.13	1.24	1.27	1.23	1.06	0.049	ns	ns	ns
On 42 d of age									
Breast meat									
Dry matter	26.86	27.43	27.04	26.84	27.57	0.191	ns	ns	ns
Protein	24.54	24.34	24.66	23.74	24.92	0.294	ns	ns	ns
Ether extract	0.97	1.13	1.11	1.27	0.79	0.152	ns	ns	ns
Ash	1.05	1.20	1.19	0.88	1.32	0.110	ns	ns	ns
Thigh meat									
Dry matter	25.12	25.07	23.89	25.94	25.46	0.236	ns	**	**
Protein	21.97	22.18	21.85	21.89	1.59	0.191	ns	ns	ns
Ether extract	1.37	1.60	1.40	1.59	1.15	0.116	ns	ns	ns
Ash	1.12	0.90	0.85	1.07	1.12	0.075	ns	ns	ns

SEM, standard error of the mean.

1) The values are means of the four replicates with two birds per treatment.

2) PC, crude protein content of starter diets; BCAA, added blends of branched-chain amino acids; N, starter diet (22% to d 15) formulated with normal crude protein; L, starter diet formulated (20% to d 15) with low crude protein; 0L:0I:0V, diet without of BCAA blends; 4L:1I:1V, diet added L-leucine, L-isoleucine, and L-valine at 1.0, 0.25, and 0.75 g/kg; 4L:1I:3V, diet added L-leucine, L-isoleucine, and L-valine at 1.0, 0.25, and 0.75 g/kg.

a,b Within a row, means with different superscripts differ significantly (p<0.05).

ns, nonsignificant; p>0.05;

* p<0.05;

** p<0.01.

**Table 6:** The meat and liver colour values and meat water holding capacity of broilers fed starter diets with different levels of protein and blends of branched-chain amino acids under hot summer conditions

Variable1)	PC2)		BCAA2)			SEM	Main effect of		
		
	N	L	0L:0I:0V	4L:1I:1V	4L:1I:3V		PC	BCAA	PC×BCAA
Breast meat									
L*	51.98	51.92	52.74	52.38	50.04	0.563	ns	ns	ns
a*	5.57	5.47	5.44	5.69	5.42	0.139	ns	ns	ns
b*	6.07	5.66	6.10^ab^	6.53^a^	4.96^b^	0.238	ns	*	ns
WHC	56.10^a^	53.13^b^	55.25	54.43	54.18	0.413	**	ns	ns
Thigh meat									
L*	59.35	60.20	60.11	60.19	59.02	0.333	ns	ns	*
a*	8.70	8.07	8.32	8.12	8.72	0.219	ns	ns	ns
b*	7.13	6.09	5.81^b^	7.55^a^	6.45^ab^	0.252	ns	*	ns
WHC	61.02^a^	59.32^b^	60.16	59.86	60.50	0.408	*	ns	ns
Liver									
L*	38.02	38.01	37.56	38.08	38.41	0.653	ns	ns	ns
a*	19.99	19.68	18.78^b^	21.40^a^	19.33^b^	0.391	ns	*	ns
b*	7.53	8.30	7.48	8.75	7.51	0.308	ns	ns	ns

SEM, standard error of the mean; WHC, water holding capacity.

1) The values are means of the four replicates with two birds per treatment.

2) PC, crude protein content of starter diets; BCAA, added blends of branched-chain amino acids; N, starter diet (22% to d 15) formulated with normal crude protein; L, starter diet formulated (20% to d 15) with low crude protein; 0L:0I:0V, diet without of BCAA blends; 4L:1I:1V, diet added L-leucine, L-isoleucine, and L-valine at 1.0, 0.25, and 0.75 g/kg; 4L:1I:3V, diet added L-leucine, L-isoleucine, and L-valine at 1.0, 0.25, and 0.75 g/kg.

a,b Within a row, means with different superscripts differ significantly (p<0.05).

ns, nonsignificant; p>0.05;

* p<0.05;

** p<0.01.

**Table 7:** The relative weight (g/100 g body weight) of the gastrointestinal tract (gut) and its segments from broilers fed starter diets with different levels of protein and blends of branched-chain amino acids under hot summer conditions

Variable1)	PC2)		BCAA2)			SEM	Main effect of		
		
	N	L	0L:0I:0V	4L:1I:1V	4L:1I:3V		PC	BCAA	PC×BCAA
On 14 d of age									
Whole gut	14.94	14.78	13.77	15.54	15.29	0.321	ns	ns	*
Proventriculus	0.82	0.80	0.74^b^	0.80^b^	0.90^a^	0.009	ns	*	ns
Gizzard	2.32	2.35	2.36	2.33	2.32	0.049	ns	ns	ns
Duodenum	1.25	1.11	1.16	1.20	1.19	0.044	ns	ns	ns
Jejunum	3.39	3.23	3.14	3.09	3.73	0.084	ns	**	*
Ileum	2.21	2.03	1.95	2.14	2.30	0.069	ns	ns	*
Large intestine	0.37	0.28	0.30	0.35	0.33	0.027	ns	ns	*
On 42 d of age									
Whole gut	7.81	7.74	7.72	7.72	7.88	0.201	ns	ns	ns
Proventriculus	0.37	0.34	0.36	0.37	0.34	0.009	ns	ns	ns
Gizzard	1.42	1.33	1.31	1.32	1.49	0.044	ns	ns	ns
Duodenum	0.53	0.50	0.50	0.55	0.50	0.018	ns	ns	ns
Jejunum	1.10	1.17	1.16^ab^	1.02^b^	1.23^a^	0.034	ns	*	ns
Ileum	0.93	0.92	0.89	0.96	0.92	0.030	ns	ns	ns
Large intestine	0.11	0.19	0.10	0.10	0.24	0.033	ns	ns	ns

SEM, standard error of the mean.

1) The values are means of the four replicates with two birds per treatment.

2) PC, crude protein content of starter diets; BCAA, added blends of branched-chain amino acids; N, starter diet (22% to d 15) formulated with normal crude protein; L, starter diet formulated (20% to d 15) with low crude protein; 0L:0I:0V, diet without of BCAA blends; 4L:1I:1V, diet added L-leucine, L-isoleucine, and L-valine at 1.0, 0.25, and 0.75 g/kg; 4L:1I:3V, diet added L-leucine, L-isoleucine, and L-valine at 1.0, 0.25, and 0.75 g/kg.

a,b Within a row, means with different superscripts differ significantly (p<0.05).

ns (nonsignificant), p>0.05;

* p<0.05;

** p<0.01.

**Table 8:** Interaction effect of different levels of protein and the branched-chain amino acids blends on relative gastrointestinal tract (gut) weight, feed intake, mortality and meat dry matter content in broilers under hot summer conditions

Variable	N0L:0I:0V1)	N4L:1I:1V1)	N4L:1I:3V1)	L0L:0I:0V1)	L4L:1I:1V1)	L4L:1I:3V1)
At 14 d of age						
Relative weights (g/100 g BW) of 2)						
Whole gut	15.09^a^	14.70^ab^	15.03^a^	12.45^b^	16.39^a^	15.60^a^
Jejunum	3.60^ab^	2.97^bc^	3.60^ab^	2.67^c^	3.20^bc^	3.89^a^
Ileum	2.32^a^	2.04^ab^	2.28^a^	1.57^b^	2.24^a^	2.33^a^
Large intestine	0.42^a^	0.42^a^	0.28^ab^	0.19^b^	0.28^ab^	0.39^ab^
DMC of thing meat (%)	25.46^ab^	24.03^b^	24.75^ab^	23.21^b^	27.01^a^	25.29^ab^
At 42 d of age						
Feed intake (g/bird/d)3)	107.65^ab^	110.38^a^	105.69^ab^	110.84^a^	103.20^b^	109.92^a^
Mortality (%)	6.25^bc^	10.16^a^	4.69^c^	9.38^a^	5.21^c^	7.81^b^
Dry matter (%)	22.71^b^	27.15^a^	25.29^ab^	24.87^b^	24.73^b^	25.32^ab^
L* value of thigh meat2)	58.17^c^	61.03^ab^	58.85^bc^	62.06^a^	59.36^bc^	59.21^bc^

BW, body weight, DMC, dry matter content.

1) N0L:0I:0V, the starter diets (22% to d 15) with the normal protein content (PC), added L-leucine, L-isoleucine, and L-valine at zero (0L:0I:0V); N4L:1I:1V, the starter diets with the normal PC added L-leucine, L-isoleucine, and L-valine at 1.0, 0.25, and 0.25 (4L:1I:1V) g/kg of diet; N4L:1I:3V, the starter diets with the normal PC added L-leucine, L-isoleucine, and L-valine at 1.0, 0.25, and 0.75 (4L:1I:3V) g/kg of diet; L0L:0I:0V, the starter diets (20% to d 15) with the low PC added L-leucine, L-isoleucine, and L-valine at zero; L4L:1I:1V, the starter diets with the low PC added L-leucine, L-isoleucine, and L-valine at 1.0, 0.25, and 0.25 (4L:1I:1V) g/kg of diet; L4L:1I:3V, the starter diets with the low PC added L-leucine, L-isoleucine, and L-valine at 1.0, 0.25, and 0.75 (4L:1I:3V) g/kg of diet.

2) The values are means of the four replicates with two birds per treatment.

3) The values are means of the four replicates with 32 birds per treatment.

a–c Within a row, means with different superscripts differ significantly (p<0.05).
